# Sphingosine-1-phosphate derived from PRP-Exos promotes angiogenesis in diabetic wound healing via the S1PR1/AKT/FN1 signalling pathway

**DOI:** 10.1093/burnst/tkad003

**Published:** 2023-05-23

**Authors:** Tianyi Chen, Peiyang Song, Min He, Shunli Rui, Xiaodong Duan, Yu Ma, David G Armstrong, Wuquan Deng

**Affiliations:** Department of Endocrinology, Chongqing Emergency Medical Center, Chongqing University Central Hospital, School of Medicine, Chongqing University, Chongqing, 400014, China; Department of Endocrinology, Chongqing Emergency Medical Center, Chongqing University Central Hospital, School of Medicine, Chongqing University, Chongqing, 400014, China; Department of Endocrinology, Chongqing Emergency Medical Center, Chongqing University Central Hospital, School of Medicine, Chongqing University, Chongqing, 400014, China; Department of Endocrinology, Chongqing Emergency Medical Center, Chongqing University Central Hospital, School of Medicine, Chongqing University, Chongqing, 400014, China; Department of Rehabilitation, The Affiliated Hospital of Southwest Medical University, Luzhou, Sichuan, 646000, China; Department of Endocrinology, Chongqing Emergency Medical Center, Chongqing University Central Hospital, School of Medicine, Chongqing University, Chongqing, 400014, China; Department of Surgery, Keck School of Medicine of University of Southern California, Los Angeles, CA 90033, USA; Department of Endocrinology, Chongqing Emergency Medical Center, Chongqing University Central Hospital, School of Medicine, Chongqing University, Chongqing, 400014, China

**Keywords:** Exosomes, Platelet-rich plasma, Diabetic foot ulcer, Wound healing, Sphingosine-1-phosphate

## Abstract

**Background:**

Sphingosine-1-phosphate (S1P), a key regulator of vascular homeostasis and angiogenesis, is enriched in exosomes derived from platelet-rich plasma (PRP-Exos). However, the potential role of PRP-Exos-S1P in diabetic wound healing remains unclear. In this study, we investigated the underlying mechanism of PRP-Exos-S1P in diabetic angiogenesis and wound repair.

**Methods:**

Exosomes were isolated from PRP by ultracentrifugation and analysed by transmission electron microscopy, nanoparticle tracking analysis and western blotting. The concentration of S1P derived from PRP-Exos was measured by enzyme-linked immunosorbent assay. The expression level of S1P receptor1–3 (S1PR1–3) in diabetic skin was analysed by Q-PCR. Bioinformatics analysis and proteomic sequencing were conducted to explore the possible signalling pathway mediated by PRP-Exos-S1P. A diabetic mouse model was used to evaluate the effect of PRP-Exos on wound healing. Immunofluorescence for cluster of differentiation 31 (CD31) was used to assess angiogenesis in a diabetic wound model.

**Results:**

*In vitro*, PRP-Exos significantly promoted cell proliferation, migration and tube formation. Furthermore, PRP-Exos accelerated the process of diabetic angiogenesis and wound closure *in vivo*. S1P derived from PRP-Exos was present at a high level, and S1PR1 expression was significantly elevated compared with S1PR2 and S1PR3 in the skin of diabetic patients and animals. However, cell migration and tube formation were not promoted by PRP-Exos-S1P in human umbilical vein endothelial cells treated with shS1PR1. In the diabetic mouse model, inhibition of S1PR1 expression at wounding sites decreased the formation of new blood vessels and delayed the process of wound closure. Bioinformatics analysis and proteomics indicated that fibronectin 1 (FN1) was closely related to S1PR1 due to its colocalization in the endothelial cells of human skin. Further study supported that FN1 plays an important role in the PRP-Exos-S1P-mediated S1PR1/protein kinase B signalling pathway.

**Conclusions:**

PRP-Exos-S1P promotes angiogenesis in diabetic wound healing via the S1PR1/protein kinase B/FN1 signalling pathway. Our findings provide a preliminary theoretical foundation for the treatment of diabetic foot ulcers using PRP-Exos in the future.

## Highlights

- Considered as the key regulator of angiogenesis and revascularization, S1P is present at a higher level in PRP-Exos than in activated supernatants of PRP.
- S1PR1 is significantly more elevated in diabetic skin than S1PR2 and S1PR3 compared with non-diabetic skin.
- PRP-Exos-S1P exerts an effective promoting effect on diabetic angiogenesis and wound healing via the S1PR1/protein kinase B/fibronectin 1 signalling pathway.

## Background

The impaired healing capacity of diabetic wounds leads to chronic wounds, amputation and even death, resulting in severe public and family burdens [1, 2]. Therefore, prompt promotion of diabetic wound healing is vital to reduce amputation and mortality events [2]. Wound healing requires complicated and integrated biological processes involving cell proliferation and migration, collagen deposition, extracellular matrix (ECM) remodelling and vascularization [3, 4]. Insufficient oxygen supply and blood flow occur in the lower-extremities of diabetic patients, contributing to microcirculation disorders, angiogenesis dysfunction and tissue formation failure [5–7]. Thus, the regeneration and remodelling of blood vessels are the key to promoting diabetic wound healing.

Both the International Work Group of Diabetic Foot (IWGDF) and our previous research indicate that plasma-rich platelet (PRP) therapy is a promising treatment for diabetic foot ulcers due to the neovascularization, anti-infection and anti-inflammatory effects [8–11]. However, the application of PRP therapy for patients with diabetic foot is limited due to the scarcity of autologous platelets and possible immune-related adverse reaction to allogeneic platelets [12]. Recently, studies involving exosomes have attracted much attention in the regeneration field. Therefore, the efficacy and mechanism of exosomes derived from PRP (PRP-Exos) for diabetic wound healing are worth exploring because of their low immunogenicity and good stability. As an ideal delivery system, exosomes bring the cargoes packaged by the phosphate bilayer to targeted sites and protect the contents from degrading and turning over [8, 13]. Moreover, accumulating evidence suggests that PRP-Exos as the condensed product of PRP, have similar functional substances but at a higher concentration [14, 15]. Based on our previous study, the isolation and identification of PRP-Exos have been successfully achieved [16]. It is well known that exosomes consist of abundant proteins, microRNAs (miRNAs) and growth factors, but the contribution of phosphate lipids is less well defined. Recently, some researchers have demonstrated that sphingosine-1 phosphate (S1P) can be separated and identified from PRP-Exos through rapid-resolution liquid chromatography and tandem mass spectrometry, and S1P was considered the key regulator maintaining the integrity of endothelial cells [17]. To investigate the role of S1P in PRP-Exos, we tested the level of S1P by enzyme-linked immunosorbent assay (ELISA) in our preliminary study. To date, some evidence suggests that vascular endothelial growth factor (VEGF), platelet-derived growth factor-BB and transforming growth factor-β released from PRP are more enriched in PRP-Exos than in supernatants of activated PRP (PRP-AS) after ultracentrifugation [18–20]. Interestingly, we found that S1P was also present at a significantly higher level in PRP-Exos than in PRP-AS.

S1P is a bioactive lipid synthesized by sphingosine kinase 1/2 and is thought to be the intracellular second messenger that transmits small molecule signals to targeted sites by binding to G protein-coupled receptors on the cell surface via S1P receptors (S1PR1–5) [21–23]. Although S1P is considered a vital phosphate lipid in the regulation of vascularization and regeneration of blood vessels [23, 24], the role and possible mechanism of S1P derived from PRP-Exos (PRP-Exos-S1P) in diabetic wound healing remain unknown. It is well known that S1PR4 and S1PR5 are expressed mainly in the nervous and immune systems, yet S1PR1–3 are highly expressed in skin tissues. Therefore, it is essential to combine S1P with S1PR1–3 for the regulation of angiogenesis and homeostasis. Based on our preliminary study, we found that S1PR1 was significantly elevated compared with S1PR2 and S1PR3 in the skin of diabetic lower extremities.

Fibronectin 1 (FN1) is an abundant protein in the basement membrane ECM that is involved in a variety of biological processes ranging from tissue survival to skin re-epithelialization and angiogenesis [25], and is suggested to be the key regulator in diabetic wound healing according to our proteomic sequencing analysis. In addition, FN1 is also an essential part of intracellular components, establishing cell adhesion, cell migration and cytoskeletal organization [26]. Bioinformatic data from the Human Protein Atlas (www.proteinatlas.org) demonstrate that the endothelial cells of human skin are rich in FN1 [27] and are present at the same expression site as S1PR1–3. Moreover, accumulating evidence suggests that S1P binding to S1PR1 is considered the key mediator activating the PI3K/protein kinase B (AKT) signalling pathway to regulate angiogenesis and vascularization [28, 29]. Some researchers have noted that FN1 has a close association with the PI3K/AKT pathway [28]. In light of the colocalization of S1PR1 and FN1, with the data from Gene Cards (www.genecards.org) in Figure S3 (see online supplementary material), we hypothesize that PRP-Exos-S1P, by binding to S1PR1, mediates the regulation of FN1 via the PI3K/AKT signalling pathway, which in turn improves the ECM of blood vessels. Accordingly, this research employed a diabetic model both *in vivo* and *in vitro* with or without PRP-Exos intervention to explore the effects on angiogenesis, wound healing and potential mechanisms. In the present study, we demonstrate that PRP-Exos-S1P promotes angiogenesis and wound healing in a diabetic model via the S1PR1/AKT/FN1 signalling pathway. Our findings provide a preliminary theoretical foundation for the clinical application of PRP-Exos for diabetic foot ulcers.

## Methods

### Activation of platelets

Whole blood (1500 ml in total) was donated by 15 healthy volunteers (age: 25 ± 5 years; gender: 8 males and 7 females). All volunteers provided written informed consent following the approval of the Ethical Committee Board of Chongqing Emergency Medical Center (number: AF/04/02.0, date: 9 June 2021). PRP was isolated by a fully automatic blood separator (CS-3000 plus; Baxer International Inc, Deerfield, IL, USA), with an average concentration of 1048 x 10^9^ platelets/l. Fresh PRP was activated by a prepared mixture (1000 IU of thrombin dry powder and 1 ml of 10% calcium gluconate solution) at a ratio of 10 : 1. The mixed liquid was shaken thoroughly and incubated at 37°C for 1 h.

### Separation of PRP-Exos

The detailed preparation protocol of PRP-Exos was described in our previous study [16]. After PRP activation finished, a series of gradient centrifugations at 500, 1500 and 2500 g for 10 min each at 4°C was conducted. Then, the supernatants were collected and passed through a 0.45 μm filter to remove the large residues. Exosome isolation was conducted by high-speed centrifugation (Optimal L-100XP, Beckman) twice for 70 min at 100,000 g. After the first phase was finished, the supernatants were collected and named PRP-AS. Then, the precipitates were washed with sterile phosphate buffered saline (PBS, pH = 7.2–7.4) and spun for another 70 min at 100,000 g. Finally, the precipitated exosomes, named PRP-Exos, were resuspended in 100 $\mu \mathrm{l}$ of sterile PBS and stored at −80°C.

### Identification of PRP-Exos

Transmission electron microscopy was used to observe PRP-Exo morphology. Nanoparticle tracking analysis was used to measure the size distribution of PRP-Exos. Exosome-specific surface biomarkers, such as cluster of differentiation 63 (CD63), flotillin and TSG101, and the platelet-specific biomarkers CD41 and calnexin were examined by western blotting.

### HUVEC culture

Human umbilical vein endothelial cells (HUVECs) were purchased from Procell Company (Wuhan, China). HUVECs were cultured in 11.1 mM glucose 1640 medium (12 633 012, Gibco) supplemented with 10% fetal bovine serum (10099141C, Gibco). After HUVECs were seeded onto culture plates, the 11.1 mM glucose medium was substituted with the 25 mM glucose 1640 medium (11.1 mM glucose 1640 medium mixed manually with glucose solution).

### Inhibitor and agonist

The AKT phosphorylation inhibitor LY294002 (S1005, Selleck) and agonist SC79 (S7863, Selleck) were used to study the PI3K/AKT signalling pathway. After HUVECs were seeded onto 6-well plates and grown to 90% confluence, the previous medium was substituted with medium mixed with LY294002 or SC79. The effects of LY294002 and SC79 were analysed by western blotting.

### ELISA

ELISA kits were used to measure the concentration of S1P (JM-0653H, JingMei) in PRP-Exos, PRP-AS and normal control groups (NC) and the concentration of VEGF-A (EK0539, Boster) in the supernatants of HUVECs transfected with FN1 siRNA (si-FN1) or negative control small RNA sequences. Before measurement of S1P in PRP-Exos, three cycles of freezing and thawing were necessary to guarantee that S1P was fully released from PRP-Exos. All the following steps were performed according to the manufacturer’s instructions.

### HUVEC transfection

The S1PR1 shRNA (shS1PR1) plasmids and si-FN1 were synthesized by Tsingke Biotechnology Company (Beijing, China). Lipofectamine™ 3000 (L3000015, Invitrogen) was used for transfection. After HUVECs were seeded onto 6-well plates and grown to 70% confluence, shS1PR1 plasmids or si-FN1 were transfected into HUVECs. All subsequent steps were performed according to the Lipofectamine^TM^ 3000 instructions. To construct a stable knockdown of S1PR1 in HUVECs, puromycin (ST551, Beyotime) was used to screen wild-type HUVECs. The effects of shS1PR1 plasmids and si-FN1 were verified by western blotting. The sequences of shS1PR1 and si-FN1 are listed in Table 1.

**Table 1:** The sequences of shS1PR1 and si-FN1

**ID**	**Forward (5′-3′)**	**Reverse (5′-3′)**
shS1PR1#1	GCCGCAGCAAATCGGACAATT	AATTGTCCGATTTGCTGCGG
shS1PR1#2	GACAACCCAGAGACCATTATG	CATAATGGTCTCTGGGTTGT
si-FN1#1	GGAGUUGAUUAUACCAUCATT	UGAUGGUAUAAUCAACUCCTT
si-FN1#2	GGUUAUAGAAUUACCACAATT	UUGUGGUAAUUCUAUAACCTT

*shS1PR1* sphingosine-1-phosphate shRNA, *si-FN1* fibronectin 1 siRNA

### HUVEC viability and proliferation

HUVEC viability was measured using a cells counting kit-8 (CCK-8) (C6005, NCM Biotech), and all subsequent steps were performed according to the CCK-8 instructions. CCK-8 was read at 450 nm absorbance using an iMark™ microplate absorbance reader (1 681 135, Bio-Rad).

HUVEC proliferation was measured using a BeyoClick™ 5-ethynyl-2′ -deoxyuridine (EdU) cell proliferation kit with Alexa Fluor 555 (C0075S, Beyotime). After HUVECs were seeded onto 12-well plates with clean and flat slides on the wells and grown to 70% confluence, PRP-Exos, PRP-AS and NC were added to the plates. The slides were removed and fixed with 4% paraformaldehyde for 20 min. All subsequent steps were performed according to the EdU kit instructions. Images were captured by an immunofluorescence (IF) microscope (ZEISS, Germany). The positive rate =cells labelled with Alexa Fluor 555/cells labelled with 4′,6-diamidino-2-phenylindole (DAPI) was used to evaluate the proliferative capacity.

### HUVEC migration

After HUVECs were seeded onto 24-well plates and grown to 90% confluence, cell scratches were made with 200 μl pipette tips, and then PRP-Exos, PRP-AS and NC were added to the plates. The results were observed within 40 h by a Lionheart FX automated microscope (Biotek, USA).

HUVECs were seeded onto the upper chambers of transwell plates (3422, Corning) at a concentration of 4 x 10^5^/ml per 100 μl of the serum-free medium. The lower chambers were filled with 10% fetal bovine serum medium mixed with PRP-Exos, PRP-AS or NC. After incubation at 37°C for 16 h, the transwell plates were removed, fixed with 4% paraformaldehyde for 20 min and then stained with crystal violet dyeing solution for 15 min. The stained upper wells were wiped off with clean cotton swabs before photos were taken using a microscope system.

### Tube formation

Tube formation experiments based on matrix gels (356 234, Corning) were conducted to evaluate the capacity of HUVECs to form blood vessels. Briefly, 70 μl liquid gels were evenly embedded in the per well of a 48-well plates on ice, and then the plates were transferred to an incubator at 37°C for 1 h. After the gel was fully solidified into a thin, transparent substance, HUVECs were seeded onto the gel at a concentration of 5 x 10^5^/ml per 100 μl and mixed with PRP-Exos, PRP-AS and NC. Tube formation was observed and recorded by microscopy within 6 h.

### Flow cytometry

Flow cytometry was used to analyse HUVEC cell cycle treated with PRP-Exos, PRP-AS and NC. All steps were performed according to the instructions of the cell cycle and cell apoptosis analysis kit (C1052, Beyotime). Measurement of the cell cycle distribution was performed using a flow cytometer (Beckman, USA).

### Q-PCR array

Total RNA was extracted with TRIzol™ reagent (15 596 018, Invitrogen), and cDNA was synthesized from 1 μg of total RNA by a high-capacity cDNA synthesis kit (4 368 814, Invitrogen). Next, real time quantitative PCR (RT-qPCR) was performed using the iTaq™ Universal SYBR Green Supermix kit (1 725 120, Bio-Rad). The results were analysed by CFX™ 96 manager (Bio-Rad, USA). Then, the expression of each mRNA was quantitated through normalization to the internal reference glyceraldehyde-3-phosphate dehydrogenase (GAPDH), and the 2^−ΔΔCT^ method was applied to determine the relative quantity. All the forward and reverse primers are listed in Table 2.

**Table 2:** Primers used in the study

**Primer**	**Specie**	**Forward (5′-3′)**	**Reverse (5′-3′)**
S1PR1	Human	TTCCACCGACCCATGTACTAT	GCGAGGAGACTGAACACGG
S1PR2	Human	CATCGTCATCCTCTGTTGCG	GCCTGCCAGTAGATCGGAG
S1PR3	Human	CGGCATCGCTTACAAGGTCAA	GCCACGAACATACTGCCCT
S1PR1	Mouse	ATGGTGTCCACTAGCATCCC	CGATGTTCAACTTGCCTGTGTAG
S1PR2	Mouse	ACAGCAAGTTCCACTCAGCAA	CTGCACGGGAGTTAAGGACAG
S1PR3	Mouse	ACTCTCCGGGAACATTACGAT	CCAAGACGATGAAGCTACAGG

*S1PR1* sphingosine-1-phosphate receptor 1, *S1PR2* sphingosine-1-phosphate receptor 2, *S1PR3* sphingosine-1-phosphate receptor 3

### Western blotting analysis

Protein was extracted from HUVECs by RIPA solution (P0013B, Beyotime) combined with phenylmethanesulfonyl fluoride (PMSF) and a phosphatase inhibitor. Then, all the protein samples were mixed with 1X SDS–PAGE protein loading buffer (P0015A, Beyotime). After all the samples were denatured at 100°C for 10 min, electrophoresis was performed using a 10% SDS–PAGE gel preparation (Bio-Rad, USA) at 80 V for 30 min and 120 V for 90 min. Then, the proteins were blotted onto a di-fluoride polyvinylidene fluoride (PVDF) membrane at 240 mA for 2 h in ice water. Next, the PVDF membrane was blocked in QuickBlock™ buffer (P0252, Beyotime) for 40 min and then incubated with diluted primary antibodies (Table 3) at 4°C overnight. After washing three times with tris buffered saline with tween (TBST), horseradish-peroxidase-conjugated (HRP-conjugated) antibodies were added to the PVDF membrane. After three washes, the immunoreactive bands were visualized using ChemiDoc Touch (Bio-Rad, USA). We used a Spectra multicolour high-range protein ladder (26 625, Thermo Scientific) as the protein ladder.

**Table 3:** Primary antibodies used in the study

**Antibody**	**ID**	**Company**	**Application**	**Dilution ratio**
FN1	sc-8422	Santa	WB, IF	1:500
CD31	AF6191	Affinity	WB, IF	1:1000
p-AKT	T40067S	Abmart	WB	1:1000
AKT1/2/3	T55561S	Abmart	WB	1:1000
VEGF-A	29 301	SAB	WB, IF	1:1000
S1PR1	37 288	SAB	WB	1:1000
S1PR2	Sc-48 356	Santa	WB	1:500
S1PR3	Sc-365 589	Santa	WB	1:500
β-Actin	T09	ZSJQ-Bio	WB	1:1000
β-Tubulin	T10	ZSJQ-Bio	WB	1:1000

*FN1* fibronectin 1, *CD31* cluster of differentiation 31, *p-AKT* phosphorylated protein kinase B, *AKT1/2/3* protein kinase B 1/2/3, *VEGF-A* vascular endothelial growth factor A, *S1PR1* sphingosine-1-phosphate receptor 1, *S1PR2* sphingosine-1-phosphate receptor 2, *S1PR3* sphingosine-1-phosphate receptor 3, *β-actin* beta actin, *β-tubulin* beta tubulin

### Animals

#### Development of the diabetic model

Wild-type C57/BL6 male mice were fed a high-fat diet (60% fat) at 3 weeks old. After 4 weeks of a high-fat diet, an intraperitoneal injection of 20 mg/kg streptozotocin (Sigma-Aldrich) mixed with citrate buffer (pH = 3.2–3.5) was administered to the mice. Before the intraperitoneal injection regimen began, fasting for 12 h overnight (with free access to water) was necessary, and injection continued for five consecutive days. After five streptozotocin injections, the fasting serum glucose was tested and recorded. A blood glucose level >16.7 mM was defined as a successful diabetic mouse model [30]. The present animal study was approved by the Animal Ethics Committee of the Chongqing Emergency Medical Center.

#### Development of the diabetic wound model

After the diabetic model was established successfully, a high-fat diet was still given until the establishment of the diabetic wound model began. All diabetic mice were anaesthetized by isoflurane inhalation. Before a full-thickness wound was made on their dorsal skin by using 6 mm punch biopsy needles, the hair on the back was cleaned fully.

#### Diabetic wound administration

**Figure 1:**
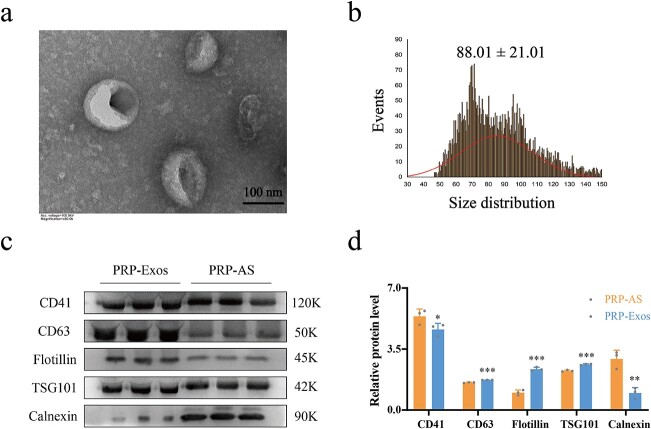
Identification of PRP-Exos. (**a**) The morphology of PRP-Exos was viewed by transmission electron microscope. (**b**) The particle size distribution of PRP-Exos was measured by nanoparticle tracking analysis; mean ± SD: 88.01 ± 21.01 nm. (**c**, **d**) Western blotting and quantitative analysis of PRP-Exos-specific markers CD63, flotillin and TSG101, and PRP-AS-specific markers CD41 and calnexin. ^*^^*^^*^p< 0.001, ^*^^*^p < 0.01, ^*^p < 0.05. PRP-Exos compared with PRP-AS. *PRP* platelet-rich plasma, *PRP-AS* activated supernatants of PRP, *PRP-Exos* exosomes derived from PRP

**Figure 2:**
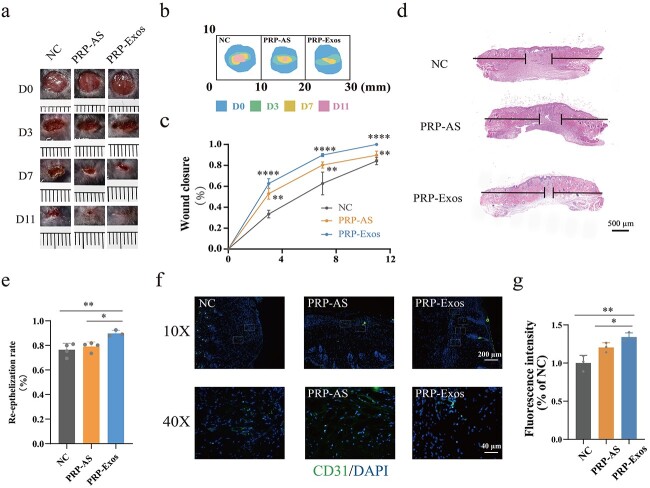
Evaluation of wound healing treated with PRP-Exos. (**a**, **b**) Representative image and mode pattern showing the process of wound closure in the NC, PRP-AS and PRP-Exos groups at days 0, 3, 7 and 11 after the operation. (**c**) Quantification of wound closure rates. ^*^^*^^*^^*^p < 0.0001, comparing PRP-Exos with NC; ^*^^*^p < 0.01, comparing PRP-Exos with PRP-AS. (**d**, **e**) Images of H&E staining and quantification of the degree of re-epithelization indicated by horizontal black lines. ^****^p< 0.0001, comparing PRP-Exos with NC; ^**^p< 0.01, comparing PRP-Exos with PRP-AS. (**f**, **g**) Images and quantification of immunofluorescence staining for CD31 labelled with Alexa Fluor 488; the cell nucleus was stained with DAPI. ^**^p < 0.01, comparing PRP-Exos with NC; ^*^p < 0.05, comparing PRP-Exos with PRP-AS. *NC* normal control, *PRP* platelet-rich plasm, *PRP-AS* activated supernatants of PRP, *PRP-Exos* exosomes derived from PRP

After the diabetic wound model was established successfully, intracutaneous injection of equivalent interventions was given at the four equal sites of the diabetic mice wound edge. All mice were randomly divided into groups as follows: (1) NC (*n* = 5); (2) PRP-AS (*n* = 5); (3) PRP-Exos (*n* = 5) (Figure 2a); (1) adenovirus-green fluorescent protein (GFP) (*n* = 6); (2) adenovirus-shS1PR1 (*n* = 6); (3) adenovirus-GFP + S1P (*n* = 6); (4) adenovirus-shS1PR1 + S1P (*n* = 6); (5) adenovirus-GFP + PRP-Exos (*n* = 6); and (6) adneovirus-shS1PR1 + PRP-Exos (*n* = 6) (Figure 6a). The process of wound healing was observed and imaged on days 0, 3, 7 and 11. The mice were sacrificed after the operation on days 0, 3, 7 and 11. Wound edges and the surrounding normal skin were harvested for further histological investigation.

#### Histology

The wound edges with partial surrounding normal skin were removed and fixed with 4% paraformaldehyde after the operation was finished on days 0, 3, 7 and 11. A series of graded ethanol was used to embed the samples in paraffin. Hematoxylin & eosin (H&E) staining and Masson trichrome staining were performed and imaged by a microscope system. Full H&E staining side scanning was conducted to observe complete and clear views. In addition, IF staining for CD31 and FN1 was performed, and cyanine-3-labelled (Cy3-labelled) goat anti-mouse IgG along with Alexa Fluor 488-labelled goat anti-rabbit IgG were used. Images were acquired using an IF microscope (ZEISS, Germany).

#### Statistics

All data are presented as the means ± standard deviations. We used the independent sample t test to evaluate the difference between the two groups. In diabetic wound groups, two-factor repeated measures analysis of variance was used to determine the difference. If three or more diabetic wound groups were compared, analysis of variance was followed by Tukey’s *post hoc* test. The graphics were made up by GraphPad Prism 8.0 software, and a *p* value < 0.05 was considered statistically significant.

## Results

### Identification of PRP-Exos

PRP-Exos exhibited typical cup- or sphere-shaped morphology (Figure 1a). Nanoparticle tracking analysis showed that PRP-Exos ranged from 50–150 nm in diameter, matching the distinctive distribution of exosomes (Figure 1b). Western blotting was used to measure the exosome surface biomarkers CD63, flotillin and TSG101 (*p* < 0.001) and the platelet-specific biomarkers CD41 and calnexin *(p* < 0.01) (Figure 1c, d). All the experiments above confirmed that exosomes were successfully isolated from PRP.

### Evaluation of wound healing treated with PRP-Exos

Digital photos and mode pictures showed the process of diabetic wound healing following treatment with PRP-Exos, PRP-AS and NC at days 0, 3, 7 and 11 (Figure 2a, b), indicating that the wound area was significantly smaller in the PRP-Exos group than in the PRP-AS or NC group at the same observation time. Quantification of wound closure rates showed that the effect of PRP-Exos was significantly greater than that of the equivalent PRP-AS or NC (*p* < 0.01) (Figure 2c). The horizontal black lines in the H&E staining pictures indicate a new epithelial layer (Figure 2d). Quantification of the degree of re-epithelization demonstrated that the effect of PRP-Exos was significantly higher than that of the equivalent PRP-AS or NC (*p* < 0.01) (Figure 2e). Masson trichrome staining for measurement of collagen deposition volume showed that there was no significant difference (*p* > 0.05) among the PRP-Exos, PRP-AS and NC groups (supplement 1, parts a and b, see online supplementary material). IF staining for CD31 showed that the formation level of new blood vessels was significantly higher in the PRP-Exos group than in the PRP-AS or NC group (Figure 2f, g). All these experiments suggested that PRP-Exos exerted significant angiogenesis-induced and re-epithelization-induced effects on the promotion of diabetic wound healing.

### Measurement of S1P in PRP-Exos and S1PR *in vivo*

**Figure 3:**
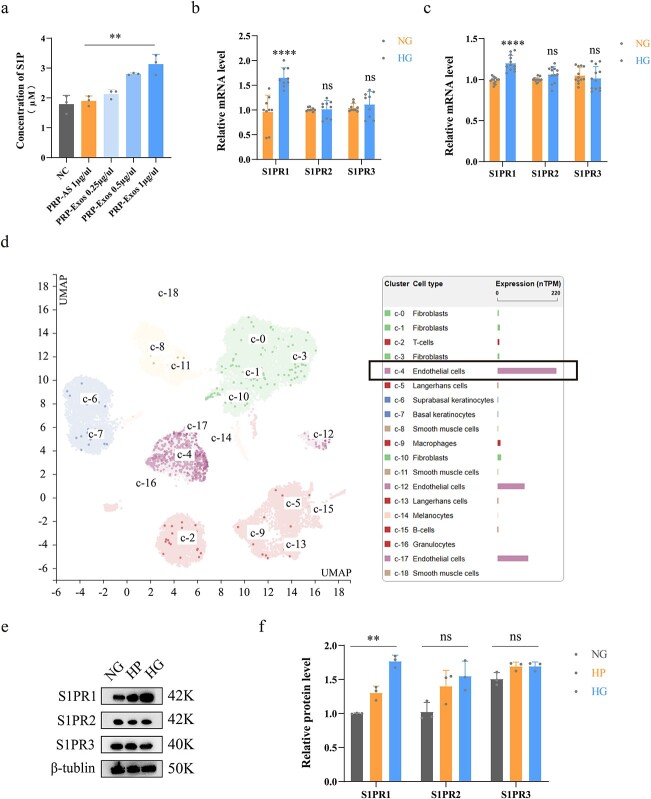
Measurement of S1P in PRP-Exos and S1PR *in vivo*. (**a**) Concentration of S1P in PRP-Exos, PRP-AS and NC groups was measured by ELISA. (**b**) Relative mRNA levels of S1PR1–3 in human skin were analysed. (**c**) Relative mRNA levels of S1PR1–3 in the mouse skin were analysed. (**d**) Data from the Human Protein Atlas verified that S1PR1 was rich in endothelial cells of human skin. (**e**, **f**) Western bloting and quantification showing the relative levels of S1PR1–3 in HUVECs; ^*^^*^^*^^*^p < 0.0001, ^*^^*^p < 0.01, *ns* no significant difference. *S1P* sphingosine-1-phosphate, *S1PR* sphingosine-1-phosphate receptor, *NG* normal glucose, *HG* high glucose, *HP* high permeation, *NC* normal control, *PRP* platelet-rich plasm, *PRP-AS* activated supernatants of PRP, *PRP-Exos* exosomes derived from PRP

Quantification of S1P in PRP-Exos was performed using ELISA. The results demonstrated that the concentration of S1P in PRP-Exos was significantly higher than that in PRP-AS (*p* < 0.01), and the level of S1P was directly proportional to the level of PRP-Exos (Figure 3a). Measurement of S1PR1–3 mRNA levels in human and mouse skin was conducted by Q-PCR. The results showed that the S1PR1 level was significantly elevated in diabetic skin compared with nondiabetic skin (*p* < 0.0001). However, S1PR2 and S1PR3 remained not significantly different in either group (Figure 3b, c). Furthermore, S1PR1 was highly expressed in the endothelial cells of human skin, as the Human Protein Atlas suggested (Figure 3d). Western blotting and quantification in wild-type HUVECs showed that S1PR1 expression was significantly higher in the high glucose group than in the normal glucose group (*p* < 0.001). However, S1PR2 and S1PR3 still remained not significantly different (Figure 3e, f).

**Figure 4:**
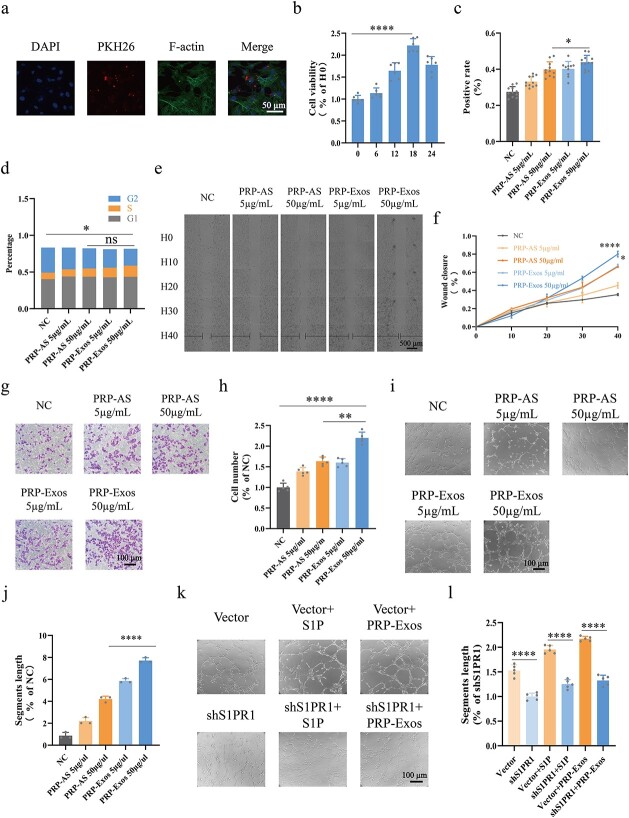
Assessment of HUVEC function. (**a**) Endocytosis assay indicated that exosomes labelled with PKH26 were carried into HUVECs within 6 h; F-actin represents the cytoskeleton. (**b**) CCK-8 assays were conducted to find the optimal time for HUVECs treated with PRP-Exos; H0 refers to zero time. (**c**) EdU assays were conducted for evaluation of HUVEC proliferation. Positive rate = (cells labelled with Alexa Fluor 555/cells labelled with DAPI) x 100%. (**d**) HUVEC cell cycle were measured by flow cytometry. Percentage of S phase cells was determined. (**e**, **f**) Cell scratch assays were observed in living cells at 0, 10, 20, 30 and 40 h. (**g**, **h**) Transwell assays were conducted for evaluation of HUVEC migration. (**i**–**l**) Tube formation assays were conducted and the segments length was used for evaluation of HUVEC tube formation capacity. ^*^^*^^*^^*^p < 0.0001, ^*^^*^p < 0.01, ^*^p < 0.05, *ns* no significant difference. *S1P* sphingosine-1-phosphate, *shS1PR1* sphingosine-1-phosphate receptor 1 shRNA, *NC* normal control, *PRP* platelet-rich plasm, *PRP-AS* activated supernatants of PRP, *PRP-Exos* exosomes derived from PRP

**Figure 5:**
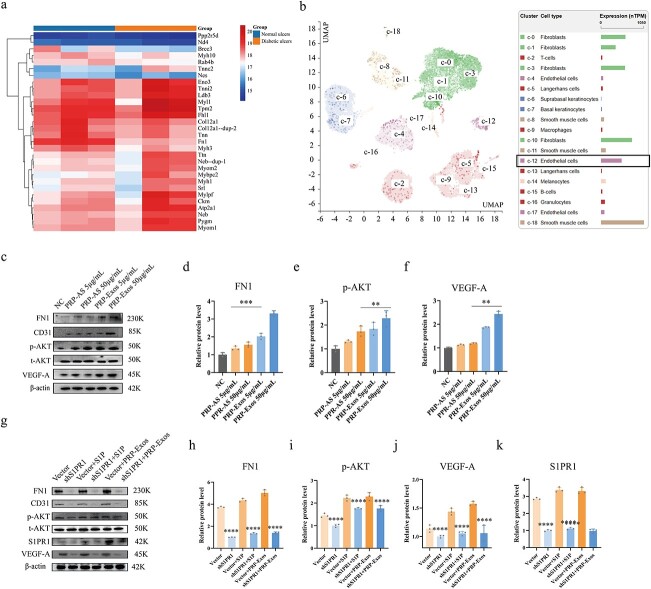
FN1 and p-AKT are involved in PRP-Exos-S1P-induced angiogenesis. (**a**) Proteomic sequencing analysis showed that FN1 was significantly lower in diabetic skin compared with nondiabetic skin. (**b**) Bioinformatic data from the Human Protein Atlas confirmed that FN1 was rich in endothelial cells of human skin. (**c**–**f**) The relative levels of FN1, p-AKT and VEGF-A in HUVECs after different treatments were measured by western blotting in the PRP-Exos 50 μg/ml *vs* PRP-AS 50 μg/ml groups. (**g**–**k**) The relative levels of FN1, p-AKT, VEGF-A and S1PR1 in HUVECs after different treatments were measured by western blotting in the shS1PR1 *vs* vector, shS1PR1 + S1P *vs* vector + S1P, shS1PR1 + PRP-Exos *vs* vector + PRPExos groups. ^*^^*^^*^^*^p < 0.0001, ^*^^*^^*^p < 0.001, ^*^^*^p < 0.01. *FN1* fibronectin 1, *p-AKT* phosphorylated protein kinase B, *t-AKT* total protein kinase B, *VEGF-A* vascular endothelial growth factor A, *S1P* sphingosine-1-phosphate, *S1PR1* sphingosine-1-phosphate receptor 1, *NC* normal control, *PRP* platelet-rich plasm, *PRP-AS* activated supernatants of PRP, *PRP-Exos* exosomes derived from PRP

**Figure 6:**
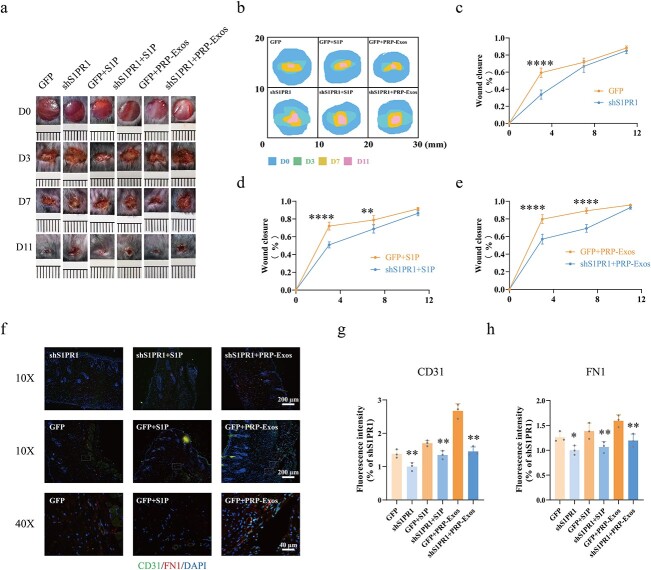
Evaluation of wound healing treated with adenovirus-shS1PR1, PRP-Exos and S1P. (**a**, **b**) Representative images and mode pattern showing the process of wound closure treated with GFP, shS1PR1, GFP + S1P, shS1PR1 + S1P, GFP + PRP-Exos, shS1PR1 + PRP-Exos at days 0, 3, 7 and 11 after the operation. (**c**–**e**) Quantification of wound closure rates in the GFP *vs* shS1PR1, GFP + S1P *vs* shS1PR1 + S1P and GFP + PRP-Exos *vs* shS1PR1 + PRP-Exos groups. (**f**–**h**) Images and quantification of immunofluorescence staining for CD31 labelled with Alexa Fluor 488, and FN1 labelled with Cy3. The cell nucleus was stained with DAPI. ^*^^*^^*^^*^p< 0.0001, ^*^^*^p < 0.01, ^*^p < 0.05. *GFP* green fluorescent protein, *S1P* sphingosine-1-phosphate, *shS1PR1* sphingosine-1-phosphate receptor 1 shRNA, *PRP* platelet-rich plasma, *PRP-Exos* exosomes derived from PRP, *FN1* fibronectin 1

**Figure 7:**
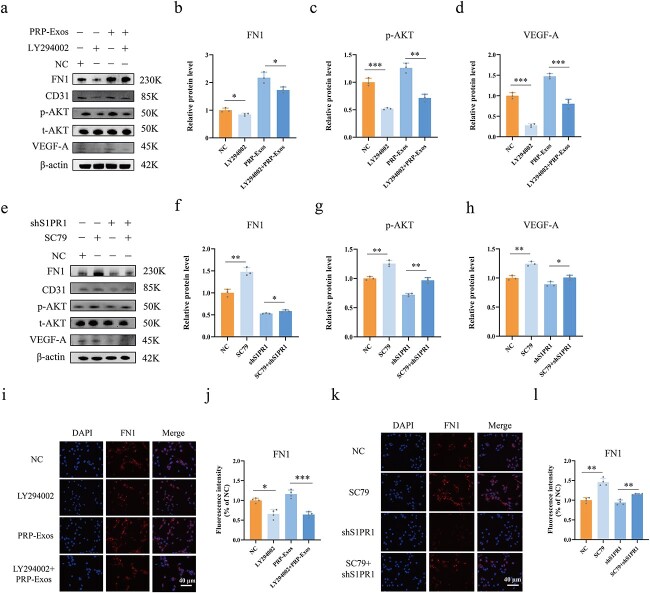
PRP-Exos-S1P regulates FN1 via the AKT signalling pathway. (**a**–**d**) The relative levels of FN1, p-AKT and VEGF-A in HUVECs after different treatments were measured by western blotting in the LY294002 *vs* NC and LY294002 + PRP-Exos *vs* PRP-Exos groups. (**e**–**h**) The relative levels of FN1, p-AKT and VEGF-A in HUVECs after different treatments were measured by western blotting in the SC79 *vs* NC and SC79 + shS1PR1 *vs* shS1PR1 groups. (**i**, **j**) Immunofluorescence for FN1 was measured in the LY294002 *vs* NC and LY294002 + PRP-Exos *vs* PRP-Exos groups. (**k**, **l**) Immunofluorescence for FN1 was measured in the SC79 *vs* NC and SC79 + shS1PR1 *vs* shS1PR1 groups. ^*^^*^^*^p < 0.001, ^*^^*^p < 0.01, ^*^p < 0.05. *LY294002* inhibitor of AKT phosphorylation, *SC79* agonist of AKT phosphorylation, *NC* normal control, *PRP* platelet-rich plasm, *PRPExos* exosomes derived from PRP, *FN1* fibronectin 1, *p-AKT* phosphorylated protein kinase B, *t-AKT* total protein kinase B, *VEGF-A* vascular endothelial growth factor A

### Assessment of HUVEC function

To explore the optimal conditions for HUVECs treated with PRP-Exos, endocytosis assays and CCK-8 assays were conducted. The data showed that PRP-Exos labelled with PKH26 (a fluorescent exosome label) were carried into HUVECs within 6 h (Figure 4a). The CCK-8 assay showed that HUVEC viability peaked at the 18th hour (*p* < 0.0001) after treatment with PRP-Exos (Figure 4b). HUVEC proliferation was analysed by EdU assay (Figure 4c), demonstrating that PRP-Exos significantly promoted HUVEC proliferation ability compared with equivalent PRP-AS *(p* < 0.05). Flow cytometry was conducted to monitor the cell cycle (Figure 4d), indicating that the percentage of S phase cells in the PRP-Exos group remained not significantly different from that in the PRP-AS group. Cell scratch and transwell assays were conducted to assess HUVEC migration. Continuous detection in living cells over 40 h revealed the process of HUVEC migration, and the black horizontal lines represent the degree of wound closure (Figure 4e). Quantification of wound closure rates showed that HUVEC migration in the PRP-Exos group was significantly promoted compared with that in the equivalent PRP-AS group (*p* < 0.05) (Figure 4f). The above result was consistent with the transwell assay results (*p* < 0.01) (Figure 4g, h). A tube formation assay was conducted to measure the blood vessel formation capacity. The results showed that the segments length of the tube in the PRP-Exos group was significantly longer than that in the PRP-AS group (*p* < 0.0001) (Figure 4i, j). After the impressive facilitation of tube formation activity induced by PRP-Exos was observed, we performed an additional study (supplement Figure S2e-h). The results showed that the segments length of the tube was significantly shorter in HUVECs treated with shS1PR1 than in those treated with vector (*p* < 0.0001) (Figure 4k, l). Consistent results (*p* < 0.0001) were observed in vector + S1P *vs* shS1PR1 + S1P and vector + PRP-Exos *vs* shS1PR1 + PRP-Exos.

### FN1 and p-AKT are involved in PRP-Exos-S1P-induced angiogenesis

According to the proteomics sequencing analysis (Figure 5a), FN1 was significantly lower in diabetic skin than in nondiabetic skin (*p* < 0.0001). The data from the Human Protein Atlas showed that FN1 was presen at a high level in the endothelial cells of human skin (Figure 5b). In light of the colocalization of S1PR1 and FN1, along with our informative work (supplement 3, part a, see online supplementary material), we hypothesized that the PRP-Exos-S1P-mediated S1PR1/AKT signaling pathway is closely related to FN1. Interestingly, western blotting showed that FN1 (*p* < 0.001), p-AKT (*p* < 0.01) and VEGF-A levels (*p* < 0.01) were significantly elevated in the PRP-Exos group compared with the PRP-AS group (Figure 5c–f). However, FN1, p-AKT, VEGF-A and S1PR1 (Figure 5g–k) were significantly lower in HUVECs treated with shS1PR1 than in those treated with vector (*p* < 0.0001). Consistent results (*p* < 0.0001) were observed in vector + S1P *vs* shS1PR1 + S1P and vector + PRP-Exos *vs* shS1PR1 + PRP-Exos.

### Evaluation of wound healing treated with adenovirus-shS1PR1, PRP-Exos and S1P

The process of diabetic wound healing after treatment with adenovirus-GFP, adenovirus-shS1PR1, adenovirus-GFP + S1P, adenovirus-shS1PR1 + S1P, adenovirus-GFP + PRP-Exos and adenovirus-shS1PR1+ PRP-Exos at days 0, 3, 7 and 11 is shown in Figure 6a, b. Quantification of wound closure rates (Figure 6c) indicated that the knockdown of S1PR1 significantly inhibited the process of wound healing compared with the GFP group at the same observation time (*p* < 0.0001). Similar outcomes were found in the GFP + S1P *vs* shS1PR1 + S1P (*p* < 0.01) and GFP + PRP-Exos *vs* shS1PR1 + PRP-Exos (*p* < 0.001) groups (Figure 6d, e).

IF staining for CD31 and FN1 was conducted for further study. The results showed that the CD31 (*p* < 0.01) and FN1 (*p* < 0.05) levels were significantly lower in the shS1PR1 group than in the GFP group (Figure 6f–h). Consistent outcomes were seen in shS1PR1 + S1P *vs* GFP + S1P and shS1PR1 + PRP-Exos *vs* GFP + PRP-Exos. Thus, we hypothesized that FN1 is involved in PRP-Exos-S1P-induced angiogenesis *in vivo*.

### PRP-Exos-S1P regulates FN1 via the AKT signalling pathway

The AKT phosphorylation agonist SC79 and inhibitor LY294002 were used for rescue experiments. Western blotting showed that FN1 (*p* < 0.05), p-AKT (*p* < 0.0001) and VEGF-A (*p* < 0.0001) were significantly lower in the LY294002 group than in the NC group. Similar levels of FN1 (*p* < 0.05), p-AKT (*p* < 0.01) and VEGF-A (*p* < 0.001) were observed in the PRP-Exos + LY294002 group compared with the PRP-Exos group (Figure 7a–d). In contrast, FN1, p-AKT, and VEGF-A were significantly elevated in the SC79 group compared with the NC group (*p* < 0.01). Similar levels of FN1 (*p* < 0.05), p-AKT (*p* < 0.01) and VEGF-A (*p* < 0.05) were observed in the shS1PR1 + SC79 group compared with the shS1PR1 group (Figure 7e–h). IF staining for FN1 in HUVECs was conducted as a supplementary experiment, and quantification of FN1 IF intensity showed consistent results with western blotting. (Figure 7i–l).

### FN1 directly regulates VEGF-A levels

**Figure 8:**
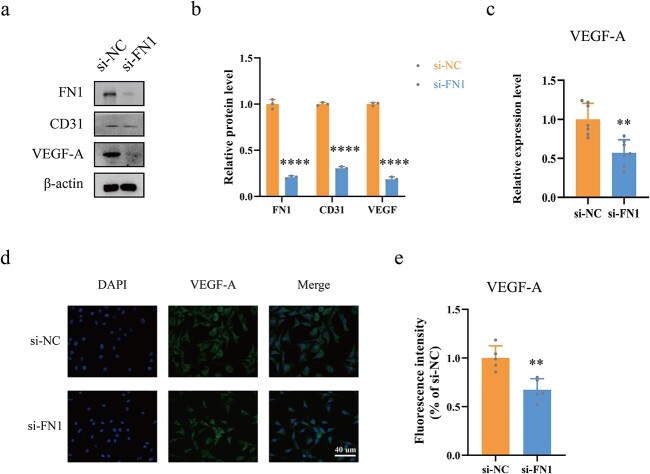
FN1 directly regulates VEGF levels. (**a**, **b**) The relative levels of VEGF-A were measured by western blotting in the si-FN1 *vs* si-NC groups. (**c**) ELISA assays were conducted for measurement of VEGF-A levels in the HUVEC supernatants after different treatments. (**d**, **e**) Immunofluorescence for VEGF-A labelled with Alexa Fluor 488 was conducted. ^*^^*^^*^^*^p < 0.0001, ^*^^*^p < 0.01. *si-NC* negative control siRNA, *si-FN1* fibronectin 1 siRNA, *FN1* fibronectin 1, *VEGF-A* vascular endothelial growth factor A

To explore the relationship between FN1 and VEGF-A, si-FN1 and si-NC were used. Western blotting and quantification (Figure 8a, b) showed that VEGF-A levels were significantly lower in the si-FN1 group than in the si-NC group. (*p* < 0.0001). In addition, ELISA was performed to test VEGF-A levels in the supernatants of HUVECs treated with si-FN1 (Figure 8c), indicating that VEGF-A levels were also significantly lower in the si-FN1 group than in the si-NC group (*p* < 0.01). Moreover, IF staining and quantification of VEGF-A (Figure 8d, e) further confirmed that VEGF-A levels were significantly lower in the si-FN1 group than in the si-NC group (*p* < 0.01).

**Figure 9:**
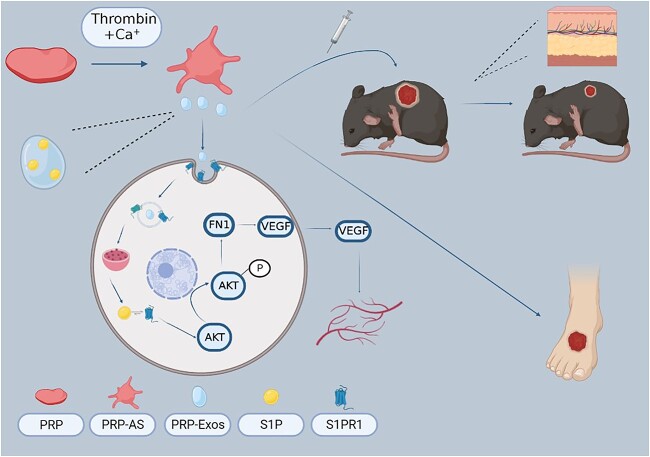
The molecular mechanism pattern was produced by Biorender (www.biorender.com). *VEGF* Vascular endothelial growth factor, *S1P* sphingosine-1-phosphate, *S1PR1* sphingosine-1-phosphate receptor 1, *PRP* platelet-rich plasma, *PRP-AS* activated supernatants of PRP, *PRP-Exos* exosomes derived from PRP, *FN1* fibronectin 1, AKT protein kinase B

## Discussion

Unlike normal wounds, diabetic wounds are stuck in oxygen-insufficient and blood-insufficient conditions and are prone to becoming chronic wounds [7, 31]. Thus, the regeneration of blood vessels in impaired wounds and the release of angiogenesis-induced cytokines are considered in the present study [7, 32]. Currently, PRP exerts an outstanding effect on diabetic wound repair, but some limitations still exist. Our study indicates that PRP-Exos promote angiogenesis and accelerate wound closure in a diabetic mouse model. Although PRP-Exos contain an abundance of content, S1P in PRP-Exos is not often mentioned. In this study, the role of S1P derived from PRP-Exos is investigated and reported for the first time. Furthermore, we elucidated the molecular mechanism of PRP-Exos in angiogenesis regulation by verifying the role of FN1 [22, 33, 34]. In the past, FN1, as a component of the ECM, was considered to have a biomechanical role in providing substrates for cell migration and adhesion via direct interactions with cell-surface receptors. Our study supports that FN1, as a downstream regulator, is involved in the PI3K/AKT pathway, exerting an angiogenesis-induced effect and directly regulating VEGF-A expression.

One of the key findings in our study is that we confirm that S1P is mainly enriched in PRP-Exos, not PRP-AS. Our quantitative analysis by ELISA for S1P in PRP-Exos makes the results more convincing. In addition, we established the shS1PR1 model under high glucose conditions both *in vivo* and *in vitro*. The S1PR1/AKT/FN1 signalling pathway responsible for delayed wound healing is explored for the first time (Figure 9). PRP-Exos-S1P has been shown to partly facilitate new blood vessel formation and wound closure by binding to S1PR1 in a diabetic wound model. Our findings verify that the knockdown of S1PR1 in endothelial cells blocks the transmission of the PRP-Exos-S1P signal almost completely. In addition, inhibited expression of S1PR1 decreases the levels of phosphorylated AKT, FN1, CD31 and VEGF-A. Interestingly, PRP-Exos-S1P partly increased the levels of p-AKT, CD31, VEGF-A and FN1 in HUVECs treated with GFP. With the assistance of the AKT phosphorylation inhibitor LY294002 and agonist SC79, we confirmed that FN1 is the downstream regulator of the PI3K/AKT pathway. Meanwhile, FN1 levels are directly related to the production of VEGF-A both inside and outside HUVECs.

In light of scarce resources and inconvenient preparation of autologous PRP, as well as the potential immune reaction to allogeneic PRP, we explored the exosomes derived from PRP for acceleration of wound closure that could overcome these weaknesses and inconvenience. Here, PRP-Exos were successfully harvested with a standard preparation method. Although there is a long way to go for the wide application of PRP-Exos in the regeneration field, PRP-Exos are still a promising and ideal ‘biological material’ due to their special structure, capacity for cross-organ and cross-species communication and convenient transportation and preservation [35, 36]. Thus, PPR-Exos combined with biomaterials have been successfully applied and reported. A specialized transforming growth factor-β-loaded PRP-Exos hydrogel could enhance ischaemic wound healing *in vivo* and *in vitro* [37]. In addition, it was also reported that PRP-Exos facilitated recovery after muscle strain injury *in vivo* [38]. A similar experiment showed that PRP-Exos exerted osteoprotective action by activating the Wnt/β-catenin pathway [20].

Although the present study suggests that PRP-Exos represent a promising therapeutic approach for diabetic wounds, another important issue still has to be addressed in future research. The specific mechanism between FN1 and VEGF-A should be further investigated. In addition, it is also worth noting that streptozotocin-induced, high-glucose animal models were used in this study, which cannot fully mimic diabetes in humans. Thus, further clinical studies should be conducted to evaluate the efficacy and safety of PRP-Exos in patients with diabetic foot ulcer.

## Conclusions

To our knowledge, this is the first time that S1P derived from PRP-Exos has been shown to promote angiogenesis and wound healing in a diabetic model by binding to S1PR1. PRP-Exos-S1P facilitates vascularization and wound closure through the S1PR1/AKT/FN1 signalling pathway. Our findings highlight the importance of using PRP-Exos as a biological therapy for diabetic wound healing.

**Abbreviations**
AKT: Protein kinase B; CCK-8: Cells counting kit-8; CD31: Cluster of differentiation 31; ECM: Extracellular matrix; ELISA: Enzyme-linked immunosorbent assay; FN1: Fibronectin 1; H&E: Hematoxylin & eosin; HRP: Horseradish peroxidase; HUVEC: Human umbilical vein endothelial cell; IF: Immunofluorescence; NC: Normal control; PBS: Phosphate-buffered saline; PDGF: Platelet-derived growth factor; PRP: Platelet-rich plasma; PRP-AS: Activated supernatants of PRP; PRP-Exos: Exosomes derived from PRP; S1P: Sphingosine-1-phosphate; S1PR: Sphingosine-1-phosphate receptor; VEGF: Vascular endothelial growth factor.

## Funding

This study was supported by the Chongqing Youth High-end Talent Studio (Grant No. ZQNYXGDRCGZS2021008), the fund of Sichuan Provincial Western Psychiatric Association’s CSPC LEADING Scientific Research Project (Grant No. WL2021002) and the Natural Science Foundation of Chongqing Municipal Science and Technology Bureau (CSTB2022NSCQ-MSX0489) awarded to WD and SR. This study is partially supported by National Institutes of Health, National Institute of Diabetes and Digestive and Kidney Diseases Award Number 1R01124789-01A1 and National Science Foundation (NSF) Center to Stream Healthcare in Place (#C2SHiP) CNS Award Number 2052578 awarded to DGA.

## Availability of data and materials

Data supporting the results of this study can be obtained from the corresponding author upon reasonable request.

## Authors’ contributions

WD and YM conceptualized the project and revised the manuscript; TC and PS conducted the experiments and drafted the manuscript; MH and SR performed the animal experiments; TC and XD participated the cells experiments; DGA reviewed and guided as a supervisor. All authors read and approved the final manuscript.

## Ethics approval and consent to participate

This study was approved by our institutional review board of Chongqing University Central Hospital (Number: AF/04/02.0, Date: 2021-06-09).

## Conflicts of interests

None declared.

## Supplementary Material

Supplement_tkad003
